# What’s important when caring for a loved one? Population-based preference weights for the Adult Social Care Outcomes Toolkit for informal carers (ASCOT-Carer) for Austria

**DOI:** 10.1007/s11136-021-02775-8

**Published:** 2021-02-17

**Authors:** Birgit Trukeschitz, Assma Hajji, Laurie Batchelder, Eirini Saloniki, Ismo Linnosmaa, Juliette Malley

**Affiliations:** 1 Research Institute for Economics of Aging, WU Vienna University of Economics and Business, Welthandelsplatz 1, D5, 1020 Vienna, Austria; 2 Personal Social Services Research Unit, University of Kent, Canterbury, UK; 3 Centre for Health Services Studies, University of Kent, Canterbury, UK; 4 Centre for Health and Social Economics, Finnish Institute for Health and Welfare (THL), Helsinki, Finland; 5 Department of Health and Social Management, University of Eastern Finland, Kuopio, Finland; 6 Care Policy and Evaluation Centre, London School of Economics and Political Science, London, UK

**Keywords:** Caregiving, Long-term care, Value, Health-related quality of life, Best–worst experiment

## Abstract

**Purpose:**

The Adult Social Care Outcomes Toolkit for informal carers (ASCOT-Carer) can be used to assess long-term care-related quality of life (LTC-QoL) of adult informal carers of persons using LTC services. The ASCOT-Carer instrument has been translated into several languages, but preference weights reflecting the relative importance of different outcome states are only available for England so far. In this paper, we estimated preference weights for the German version of the ASCOT-Carer for Austria and investigated the value people place on different QoL-outcome states.

**Methods:**

We used data from a best–worst scaling (BWS) experiment and estimated a scale-adjusted multinomial logit (S-MNL) model to elicit preference weights for the ASCOT-Carer domain-levels. Data were collected using an online survey of the Austrian general population (*n* = 1001).

**Results:**

Top levels in the domains of ‘Space and time to be yourself’, ‘Occupation’ and ‘Control over daily life’ were perceived as providing the highest utility, and states with high needs in the same domains seen as particularly undesirable. ‘Personal safety’ was the only domain where levels were roughly equidistant. In all other domains, the difference between the top two levels (‘ideal state’ and ‘no needs’) was very small.

**Conclusion:**

The paper provides preference weights for the German version of ASCOT-Carer to be used in Austrian populations. Furthermore, the results give insight into which LTC-QoL-outcomes are seen as particularly (un)desirable, and may therefore help to better tailor services directed at informal carers and the persons they care for.

## Background

Informal care (care and support provided by relatives or non-kin) is a major resource in enabling people with long-term care (LTC) needs to age in place [1]. Informal care, however, is not available for free; it comes with indirect costs [2], on, for example, informal carers’ health [3], well-being [4] and employment opportunities [5], and direct costs, such as their time and resources spent providing care [6]. In aging societies, limiting direct and indirect costs on family and non-kin carers and improving their health and quality of life (QoL) is key for LTC policy to ensure the long-run availability of informal care. In line with this, European countries, having recognised the role of informal carers and the impact of the caring role on their lives, have started to set up support systems to assist caring of family members and, but less commonly, of non-kin [7].

LTC policies differ in their logic for providing support to informal carers depending on whether providing care for care-dependent adults is considered a public responsibility or mainly delegated to family members, leading to different service types and arrangements [8]. Traditionally, the Austrian LTC system has relied heavily on the availability and provision of informal care. Benefits and services for care-dependent people also aim implicitly to reduce care burden. More recently, efforts have been increased to target support directly at informal carers; however, take-up is generally low [9]. In order to support Austrian informal carers in their caring roles, a more in-depth understanding of both the QoL-impact of publicly supported services and the desirability or preference of certain carer-relevant QoL-outcomes is needed.

A variety of instruments have been developed to measure carers’ living conditions and QoL, such as the Carer Experience Scale (CES) [10], the Adult Carers Quality of Life questionnaire (AC-QoL) [11] and the Zarit Burden Interview (ZBI) [12]. These instruments have contributed to better understand living conditions of informal carers but they do not directly capture the *effects* of service provision on informal carers’ QoL, nor do they account for the value of QoL-states. Such preference-weighted measures are mainly found for health-related QoL (e.g. EQ-5D [13] and Health Utility Indexes (HUI 2 and 3) [14]), for more general well-being (Quality of Well-Being Scale (QWB) [15]), and for QoL-impact of informal care (CarerQoL-7D [16]). The Adult Social Care Outcome Toolkit for informal Carers (ASCOT-Carer), however, directly measures QoL-effects of LTC-related services on informal carers’ QoL, irrespective of whether those services are provided to the person they care for or directly to the informal carer. The measure is preference-weighted, meaning it accounts for people’s value of different QoL-states when caring for a relative or friend. Understanding people’s preferences is particularly useful for economic evaluations aiming to better allocate scarce resources [17] as knowledge about the importance of different health or QoL-states enables the evaluation of trade-offs and improves decision-making [18].

The ASCOT-Carer instrument comprises seven QoL-domains (‘Occupation’, ‘Control over daily life’, ‘Looking after yourself (Self-Care)’, ‘Personal safety’, ‘Social participation and involvement’, ‘Space and time to be yourself’, ‘Feeling supported and encouraged’) with four outcome levels each (‘ideal state’, ‘no needs’, ‘some needs’, and ‘high needs’). Preference weights reflecting the relative importance of different outcomes in each of the domains allow for a more accurate representation of the total score [19].

The ASCOT-Carer was originally developed in England and has been translated into several languages, including German. The German version has been validated and shows good measurement properties [20]. So far, preference weights for the ASCOT-Carer instrument have been published only for England [21]. However, preferences may vary across countries due to institutional differences and different LTC systems [22–24]. In order for the ASCOT-Carer instrument to be used in evaluations in different target populations, country-specific preference weights are required to adequately reflect the relative importance of QoL-states in these countries.

This paper aims to provide preference weights for the German version of the ASCOT-Carer instrument for use in Austria, generated from a best–worst scaling (BWS) experiment, and to shed light on the relative importance of different ASCOT-Carer QoL-outcome-states. Using preference weights to calculate the ASCOT-Carer score, representing LTC-related QoL (LTC-QoL) for informal carers, enhances the informative content of the measure by better reflecting the actual value of the QoL-state in a domain. Through the elicitation of preference weights, we also identify areas in which unmet needs are seen as particularly bad and highlight QoL-states in areas of carers’ lives that are seen as particularly desirable.

## Methods

### Data collection using a best–worst experiment design

Methods to measure preferences comprise of rating, ranking and choice-based methods, with choice-based methods given priority [18]. Within choice-based methods, BWS provides a cognitively less challenging alternative to elicit preferences compared to discrete choice experiments (DCEs) [25]. BWS designs allow for the inclusion of a larger number of attributes or domains without creating tasks sets that are highly demanding, or requiring a very large sample size [26]. This makes them more feasible for use with the ASCOT-Carer instrument, which has 28 (7 × 4) domain-levels in total. BWS uses data generated from choice sets asking respondents to choose ‘best’ and ‘worst’ elements out of a list of items. Therefore, they also elicit additional information regarding least preferred options compared to a traditional DCE [27]. We used a ‘profile case’ (type 2) best–worst experiment [28] consisting of choice sets showing the seven attributes (ASCOT-Carer domains) at different levels. The profile case of BWS asks respondents to compare individual attribute-levels (instead of full profiles, as they would in a traditional DCE) [21].

The experimental plan followed the approach employed for eliciting preference weights for the English ASCOT-Carer [21]. We used an orthogonal main effects plan (OMEP) to design the choice sets and a fractional-factorial design to reduce the full factorial (4^8 scenarios) to 32 scenarios. These were grouped into blocks of 8 and each respondent was randomly assigned to one of the blocks. To ensure that the respondents would understand the tasks and explanations in the survey, we performed pre-tests using cognitive interviews (think-aloud method) prior to the data collection.

**Fig. 1:**
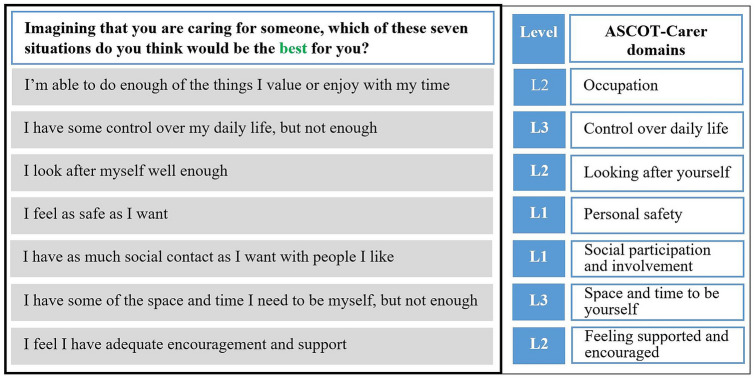
Illustration of a choice task (choice set) using the ASCOT-Carer domains. *Note*: Levels and ASCOT-Carer domain names were added for illustration and were not displayed in the survey (L1 = ideal state; L2 = no needs; L3 = some needs; L4 = high needs), the experiment used the German version of the ASCOT-Carer instrument; the Figure displays the wording of the original English instrument [29]

In the BWE experiment, respondents were asked to put themselves in the imaginary situation of being someone’s informal carer and presented with eight consecutive best–worst tasks. Each task was comprised of QoL-statements from the seven ASCOT-Carer domains (Fig. 1). In each task, respondents chose the ‘best’, ‘worst’, ‘second best’ and ‘second worst’ QoL-situations, meaning a total of 32 sequential choices. The positioning of the ASCOT-Carer domain-statements was randomised across individuals in order to prevent positioning effects from distorting preference weights for individual domain levels.

The BWS experiment was part of an online survey collecting sociodemographic characteristics, self-perceived health on a five-point scale, experience with care (including help or support provided in the last month), current QoL using the ASCOT-Carer instrument, and data on the understanding of the choice tasks.

A meta-analysis has found no significant differences between patient and general population preferences [30]. As a societal perspective is generally recommended for economic evaluations [31], a general population sample was used. Respondents were recruited via an online panel managed by Research Now (now Dynata). Data were collected in July and August 2016. The target sample size was 1000, with quotas for sex, age group and region (nine Austrian ‘Laender’) set to match national statistics.

### Statistical analysis

The statistical model underlying BWS is based on the notion that the relative choice probabilities of items reflect their distance on the latent utility scale, and relative item utility can be inferred from these probabilities [25]. Austrian preference weights for the ASCOT-Carer instrument were obtained by using a scale-adjusted multinomial logit model (S-MNL). We estimated utilities for all domain-levels on a common scale, using the item with the lowest perceived utility as a reference point. We accounted for scale heterogeneity (differences in error variance between respondent groups [32]) as well as positioning effects (heterogeneity in choice probabilities related to the placement of the items in the list [21, 33]). As we did not aim to investigate differences in preferences in sub-groups, taste heterogeneity was not analysed in detail, but was used to correct for lack of sample representativeness. Following the approach by Burge, Potoglou, Kim, and Hess [34] and Huynh, Coast, Rose, Kinghorn, and Flynn [35], group-specific coefficients were weighted by applying the correct population proportions. The estimations were performed in Alogit (2017) and Biogeme [36], using robust sandwich estimators to account for the repeated nature of the tasks. For an in-depth description of the modelling approach and study design, see Batchelder et al. [21] and Hajji et al. [37].

## Results

### Sample descriptives

The sample comprised 1001 respondents. Persons with unrealistically short completion times for the BWS experiment (less than 4.5 min) were dropped during the data collection period and sampling was continued until the target number of 1000 respondents was reached. Tables 1 and 2 give an overview of the sample. Table 1 shows sample descriptives and corresponding national distributions. Sample characteristics defined by quotas (age, sex and region) mirror the national distributions. Persons with lower education were under-represented in the sample, as were lower income groups (deciles 1–5). Since deviations for both variables were by more than 10 percentage points, we accounted for this in the analysis.

**Table 1:** Sample descriptives I: comparison to national population (*n* = 1001). *Source* WU, EXCELC B/W-C AUT 2016 (*n* = 1001), [38, 39]

	Sample		National population	
	Frequency	Percent	Frequency	Percent
*Gender*				
Male	489	48.85	3,511,968	48.64
Female	512	51.15	3,708,522	51.36
*Age*				
18–34 years	263	26.27	1,941,693	26.9
35–54 years	380	37.96	2,554,443	35.4
55 years and over	358	35.76	2,724,354	37.7
*Region*				
Burgenland	33	3.3	244,753	3.39
Carinthia	62	6.19	468,744	6.49
Lower Austria	198	19.78	1,368,348	18.95
Upper Austria	179	17.88	1,193,948	16.54
Salzburg	60	5.99	449,813	6.23
Styria	159	15.88	1,035,580	14.34
Tyrol	67	6.69	611,991	8.48
Vorarlberg	27	2.7	311,288	4.31
Vienna	216	21.58	1,536,025	21.27
*Education*				
Lower secondary and below	74	7.48	1,644,452	24.60
Upper secondary	514	51.97	3,675,949	54.98
Short-cycle tertiary and post-secondary	205	20.73	716,501	10.72
Tertiary (BA, MA, PhD or equivalent)	196	19.82	648,530	9.70
Missing	12		535,058	
Total	1001	100.0	7,220,490	100.0
*Disposable household income*				
Deciles 1–5	344	39.49	1,880,895	50.00
Deciles 6–10	527	60.51	1,880,895	50.00
Prefer not to say	130			
Total	1001	100.0	3,761,790	100.0

**Table 2:** Sample descriptives II: health and care experiences (*n* = 1001). *Source* WU, EXCELC B/W-C AUT 2016 (*n* = 1001)

	Sample	
	Frequency	Percent
*Self-perceived health*		
1. Very good	208	20.78
2. Good	476	47.55
3. Fair	238	23.78
4. Bad	68	6.79
5. Very bad	11	1.10
*Experience with care*		
1. Yes, I have personal experience	48	4.80
2. Yes, my partner	45	4.50
3. Yes, my parents	226	22.58
4. Yes, one of my children	10	1.00
5. Yes, one of my siblings (brother/sister	15	1.50
6. Yes, another relative or friend	226	22.58
7. Yes, an acquaintance, colleague or neighbour	40	4.00
8. No experience with long-term care needs	377	37.66
b. Don't know	14	1.40
*Provided help or support in the last month*		
0. No	799	79.82
1. Yes	202	20.18
Total	1001	100.0

Table 2 provides further details of the sample addressing health and care experiences. Overall, the self-assessed health status of the respondents was fairly good. Most respondents (68%) had a ‘very good’ or ‘good’ self-assessed health, only 1% of respondents listed their health as ‘very bad’. Most of the participants (60%) had had some prior experience with care (personal or through someone in their environment). When it comes to providing informal care, 20% of the respondents had stated they had provided informal help or support to someone in the month prior to the survey.

### Results of the S-MNL model for Austria

Table 3 shows estimation results from the S-MNL model accounting for scale heterogeneity and positioning effects. Coefficients for all attribute-levels were estimated in reference to ‘Space and time to be yourself’ at level 4 (‘I don’t have any space or time to be myself’). Coefficients represent the relative values assigned to the QoL-states and decreased monotonically within each domain, with level 1 having the highest value and level 4 the lowest. This was the case for all domains except for ‘Feeling supported and encouraged’, where level 2 (‘I feel I have adequate encouragement and support’) was rated slightly, but not significantly, higher than level 1 (‘I feel I have the encouragement and support I want’), indicating that the two states were valued similarly. In order to account for this and maintain consistency, we estimated a joint coefficient for level 1 and level 2 in this domain.

**Table 3:** S-MNL estimation results for Austria. *Source* WU, EXCELC B/W-C AUT 2016 (*n* = 1001)

Domain levels	Coef	s.e (rob.)	*p* value (rob.)	Pairwise significance test	
				*z* ratio	*p* value
*Occupation*					
1. I’m able to spend my time as I want, doing things I value or enjoy	3.53	0.24	< 0.001	0.46	0.32
2. I’m able to do enough of the things I value or enjoy with my time	3.38	0.22	< 0.001	8.33	< 0.001
3. I do some of the things I value or enjoy with my time, but not enough	1.34	0.10	< 0.001	8.92	< 0.001
4. I don’t do anything I value or enjoy with my time	0.36	0.05	< 0.001		
*Control over daily life*					
1. I have as much control over my daily life as I want	3.33	0.23	< 0.001	0.82	0.21
2. I have adequate control over my daily life	3.08	0.21	< 0.001	6.07	< 0.001
3. I have some control over my daily life, but not enough	1.64	0.12	< 0.001	10.10	< 0.001
4. I have no control over my daily life	0.37	0.05	< 0.001		
*Looking after yourself*					
1. I look after myself as well as I want	2.82	0.19	< 0.001	0.26	0.40
2. I look after myself well enough	2.75	0.19	< 0.001	8.67	< 0.001
3. Sometimes I can’t look after myself well enough	0.99	0.09	< 0.001	6.05	< 0.001
4. I feel I am neglecting myself	0.43	0.0493	< 0.001		
*Personal safety*					
1. I feel as safe as I want	2.56	0.17	< 0.001	3.66	< 0.001
2. Generally I feel adequately safe, but not as safe as I would like	1.78	0.12	< 0.001	4.55	< 0.001
3. I feel less than adequately safe	1.10	0.08	< 0.001	3.68	< 0.001
4. I don’t feel at all safe	0.71	0.06	< 0.001		
*Social participation and involvement*					
1. I have as much social contact as I want with people I like	2.94	0.20	< 0.001	1.35	0.09
2. I have adequate social contact with people	2.58	0.18	< 0.001	6.23	< 0.001
3. I have some social contact with people, but not enough	1.34	0.10	< 0.001	8.24	< 0.001
4. I have little social contact with people and feel socially isolated	0.45	0.05	< 0.001		
*Space and time to be yourself*					
1. I have all the space and time I need to be myself	3.50	0.24	< 0.001	0.34	0.37
2. I have adequate space and time to be myself	3.39	0.23	< 0.001	6.67	< 0.001
3. I have some of the space and time I need to be myself, but not enough	1.69	0.12	< 0.001	14.32	< 0.001
4. I don’t have any space or time to be myself	0.00				
*Feeling supported and encouraged*					
1. I feel I have the encouragement and support I want	2.71	0.18	< 0.001		
2. I feel I have adequate encouragement and support	2.71	0.18	< 0.001	7.39	< 0.001
3. I feel I have some encouragement and support, but not enough	1.20	0.09	< 0.001	7.69	< 0.001
4. I feel I have no encouragement and support	0.41	0.05	< 0.001		
*Positioning effects*					
Position 1 (best)	0.00				
Position 2 (best)	−0.10	0.03	< 0.001		
Position 3 (best)	−0.18	0.03	< 0.001		
Position 4 (best)	−0.27	0.03	< 0.001		
Position 5 (best)	−0.32	0.03	< 0.001		
Position 6 (best)	−0.36	0.03	< 0.001		
Position 7 (best)	−0.40	0.03	< 0.001		
*Scale parameters*					
Understanding of the tasks: not understood	0.70	0.04	< 0.001		
Respondent age: 35 and over	0.80	0.04	< 0.001		
Completion time: slower (1st quartile^a^ to max)	1.66	0.10	< 0.001		

© University of Kent: The ASCOT response options are reproduced with permission from the University of Kent. All rights reserved

Scale parameters for the reference groups are set to 1, *p* values were adjusted accordingly

Note that these coefficients are not corrected for sample non-representativeness, the corrected final weights are reported in section ‘Preference weights for the ASCOT-Carer measure for Austria’

^a^1st quartile threshold: 7 min

All coefficients were significantly different from the reference category, indicating that all other ASCOT-Carer states are valued as having a higher utility than ‘Space and time to be yourself’ at level 4 (‘I don’t have any space or time to be myself’). In all domains, except for ‘Safety’, level 1 and 2 coefficients were not significantly different from each other at the 5% level. All other pairwise comparisons (i.e. comparisons between level 2 and 3 as well as between level 3 and 4) showed significant differences between the levels.

Coefficients for positioning effects for best choices were all negative, significantly different from the reference category (position 1, i.e. the uppermost item in the list) and increasing in magnitude. This indicates that the lower an item was in the list, the less likely it was to be picked as the ‘best’ choice compared to the uppermost item. No significant comparable effect was found for ‘worst’ choices, hence positioning coefficients for worst choices were not included in the final model.

We used scale heterogeneity analyses to correct for differences in error variance for respondent groups. Scale parameters are inversely related to the error variance and were set to 1 for the reference group in the model. Values higher than 1 indicated smaller error variance than the reference group and vice versa. Persons who had not understood the tasks at all or only understood the tasks sometimes showed higher error variance, as did respondents aged 35 and over and persons with faster completion times (minimum to quartile 1).

### Preference weights for the ASCOT-Carer measure for Austria

To obtain the preference weights, coefficients from Table 3 were adjusted based on results from the taste heterogeneity analysis where needed, and rescaled so that the range of possible total ASCOT-Carer scores was between 0 and 1 [21].

**Fig. 2:**
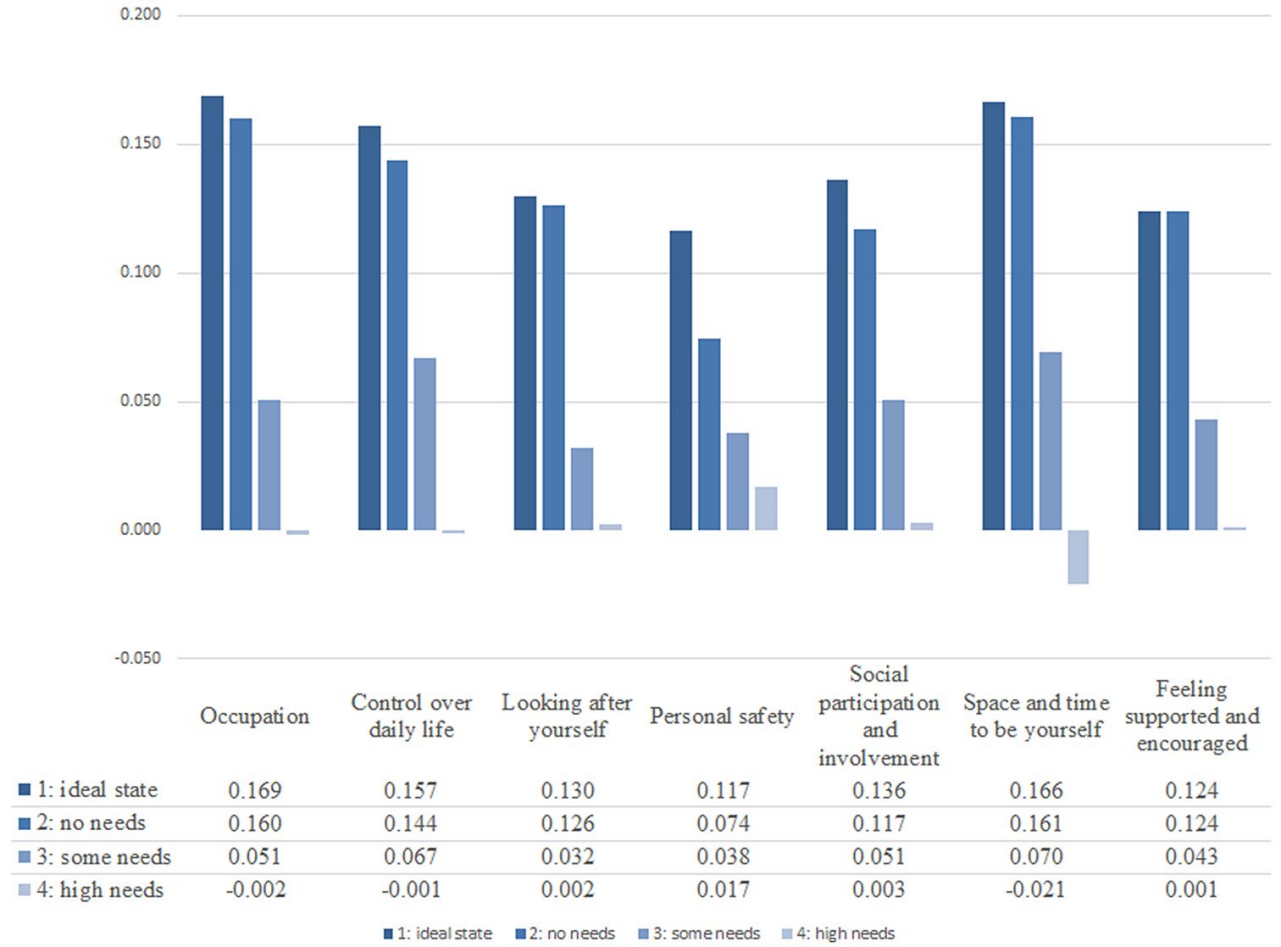
Final preference weights for the ASCOT-Carer instrument for Austria. *Source*: WU, EXCELC B/W-C AUT 2016 (*n* = 1001)

Figure 2 shows the final preference weights for the German ASCOT-Carer to be used in Austria. The highest rated QoL-state was ‘Occupation’ at level 1 (‘I’m able to spend my time as I want, doing things I value or enjoy’), followed by ‘Time and space to be yourself’ at level 1 (‘I have all the space and time I need to be myself’). Experiencing high needs (level 4) in the domain ‘Space and time to be yourself’ (‘I don’t have any space or time to be myself’) was perceived as most undesirable, followed by level 4 states in the domains ‘Occupation’ (‘I don’t do anything I value or enjoy with my time’) and ‘Control over daily life’ (‘I have no control over my daily life’).

Levels 1 (‘ideal state’) and 2 (‘no needs’) did not statistically differ, whereas there is a steep drop in perceived utility between levels 2 (‘no needs’) and 3 (‘some needs’) as well as between levels 3 (‘some needs’) and 4 (‘high needs’) for all domains except for ‘Personal safety’, where levels are roughly equidistant.

## Discussion

Supporting informal carers has been recognised as key for sustainable care arrangements [40]. Thus, information on effects of service provision and preferences is relevant for designing carers’ support to positively impact on people’s lives and for comparing policy outcomes across countries.

This paper provided preference weights for the German version of the ASCOT-Carer instrument for use in Austria. The results suggest that increasing informal carers’ ability to have enough time for themselves, pursue meaningful activities and occupation, and have control over their lives should be the focus of interventions that aim to support informal carers of adult persons in Austria. For all seven QoL-domains, the results for Austria indicate that policies for informal carers who already experience high or some needs could make a substantial difference to their lives. Policies would be particularly valued for informal carers who lack sufficient or any opportunity to relax and ‘switch off’ from their caring role and responsibilities (as measured by the domain ‘Space and time to be yourself’). This analysis identified areas of life where unmet needs will be perceived as unacceptable by the Austrian population, as well as areas of life where ideal outcomes are seen as particularly desirable. Understanding people’s preferences thus enables LTC policymakers to better allocate scarce resources to target specific goals (e.g. improving carers’ control over their daily life) or groups of carers (e.g. persons with very little space and time for themselves).

As expected, preference weights decreased monotonically within domains from level 1 (‘ideal state’) to level 4 (‘high needs’), except for ‘Feeling supported and encouraged’, where level 1 and level 2 were estimated jointly. Differences between levels 1 (‘ideal state’) and 2 (‘no needs’) were small and usually not significant, indicating a noticeable drop in perceived utility only incurs past a certain point (differences are large when comparing an ‘OK’ (‘no needs’) state to a state with ‘some needs’, but not when comparing an ‘ideal state’ to an ‘OK’ state). The ‘Personal Safety’ domain was an exception in that all level-differences were significant and comparable in size, indicating that each decrease in LTC-QoL in this domain is noticeable.

In the English analysis of preference weights for the ASCOT-Carer, the highest-rated states were ‘Occupation’ at level 1 (same as Austria) and ‘Control over daily life’ at level 1 (4th highest in Austria). The lowest-rated states were ‘Control over daily life’ at level 4 (3rd lowest in Austria) and ‘Occupation’ at level 4 (same as Austria) [21]. An accurate and detailed country comparison of population-based preferences for QoL-states when caring for an older adult requires pooling of country data and poses an interesting path for future research in this area.

The study has some limitations. First, the analysis used an online panel, which by definition will not include groups with less access to the Internet. Second, we used quota sampling and adjusted for deviations in observed characteristics not captured by the quotas by weighting coefficients with the respective population proportions. Although quota sampling is widely used, as a non-probability sampling technique, it does not guarantee representativeness on unobserved characteristics. Since we were interested in correctly estimating the relative relationship between coefficients, we made adjustments where we found these relationships to differ for groups that were under-represented. However, as we cannot correct for unobserved characteristics or characteristics whose distribution in the target population is unknown. Furthermore, there is a debate about whether the S-MNL model is suited to disentangling different sources of heterogeneity in the choice data [41]. As the focus of the paper was not on heterogeneity, which was only controlled for in order to improve model fit and correct for the lack of sample representativeness, this should not impact upon the results. It should, however, be noted that further analyses looking into heterogeneity in more detail might benefit from alternative modelling approaches [42].

The preference weights for the ASCOT-Carer instrument presented here can be useful for studies investigating informal carers’ LTC-QoL and the impact of services on carers’ LTC-QoL in Austria. They allow for a more accurate representation of people’s service-related outcomes, given that outcome states in different QoL-domains are not always valued equally and levels within domains are not necessarily equidistant. The availability of preference weights makes the ASCOT-Carer instrument better suited for use in economic evaluations of service outcomes and helps to understand informal carers’ situations that are seen as particularly (un-)desirable. In addition, future research comparing preference weights obtained for different countries, e.g. preferences for Austria and England, could give insights into country-differences in the relative importance of QoL-states when caring for a family member or non-kin.

## Data Availability

The data that support the findings cannot be made publicly available as data sharing is not covered by the informed consent.
